# Reducing DCO registrations through electronic matching of cancer registry data and routine hospital data

**DOI:** 10.1054/bjoc.1999.0985

**Published:** 2000-01-18

**Authors:** A M Pollock, N Vickers

**Affiliations:** Health Policy and Health Services Research Unit, School of Public Policy, University College London, London, WC1H 9EZ, UK; Department of Public Health Sciences, St George's Hospital Medical School, Cranmer Terrace, London, SW17 0RE, UK

## Abstract

The Thames Cancer Registry (TCR) has registered a high proportion of tumours from death certificate information only (DCO) registrations. This paper describes the results of a study set up to establish whether this proportion could be reduced by linking cancer registrations with routine hospital data from the Hospital Episodes Statistics (HES) data set using computerized matching. A total of 67 752 registrations were identified from the TCR. Matches were found in the HES data set for 66%. The proportion of cases retrieved for each tumour site was: 72% for colorectal cancer; 62% for cancer of the lung, trachea or bronchus; and 65% for female breast cancer. For all three tumour sites the proportion of matches found for patients registered from hospital case notes was higher than the proportion found for patients registered as DCOs (*P*< 0.0001 for all three tumour sites). Among matched DCO cases, 58% had at least one procedure recorded. DCO rates might be reduced by as much as 43% (from 17% of total registrations to less than 10%) for the three most common cancers if the method of electronic matching outlined here was used. Younger age groups, prognosis of tumour site and residence in North Thames region were all positively associated with successful matching (*P*< 0.0001 in all three cases). Many matched DCO cases were found to have had more than one admission for cancer. Among ordinary in-patient admissions, admissions to patients ratios of 1.5, 1.4 and 1.9 were found for colorectal, lung and breast cancers respectively. Of 5190 matched DCOs a procedure was recorded for 3013 (58%). HES data offer a useful aid to follow-up of case notes on patients identified to the registry by death certificates. Doubts about the completeness and accuracy of HES data mean case notes must remain the ‘gold standard’. © 2000 Cancer Research Campaign


*British Journal of Cancer* (2000) **82**(3), 712–717
© 2000 Cancer Research Campaign
DOI: 10.1054/ bjoc.1999.0985, available online at http://www.idealibrary.com on IDEAL®

### Reducing DCO registrations through electronic matching of cancer registry data and routine hospital data

**AM Pollock[1] and N Vickers[2]**

[1]Health Policy and Health Services Research Unit, School of Public Policy, University College London, London WC1H 9EZ, UK; [2]Department of Public Health Sciences, St George's Hospital Medical School, Cranmer Terrace, London SW17 0RE, UK

**Summary** The Thames Cancer Registry (TCR) has registered a high proportion of tumours from death certificate information only (DCO) registrations. This paper describes the results of a study set up to establish whether this proportion could be reduced by linking cancer registrations with routine hospital data from the Hospital Episodes Statistics (HES) data set using computerized matching. A total of 67 752 registrations were identified from the TCR. Matches were found in the HES data set for 66%. The proportion of cases retrieved for each tumour site was: 72% for colorectal cancer; 62% for cancer of the lung, trachea or bronchus; and 65% for female breast cancer. For all three tumour sites the proportion of matches found for patients registered from hospital case notes was higher than the proportion found for patients registered as DCOs ($P < 0.0001$ for all three tumour sites). Among matched DCO cases, 58% had at least one procedure recorded. DCO rates might be reduced by as much as 43% (from 17% of total registrations to less than 10%) for the three most common cancers if the method of electronic matching outlined here was used. Younger age groups, prognosis of tumour site and residence in North Thames region were all positively associated with successful matching ($P < 0.0001$ in all three cases). Many matched DCO cases were found to have had more than one admission for cancer. Among ordinary in-patient admissions, admissions to patients ratios of 1.5, 1.4 and 1.9 were found for colorectal, lung and breast cancers respectively. Of 5190 matched DCOs a procedure was recorded for 3013 (58%). HES data offer a useful aid to follow-up of case notes on patients identified to the registry by death certificates. Doubts about the completeness and accuracy of HES data mean case notes must remain the 'gold standard'. © 2000 Cancer Research Campaign

The Thames Cancer Registry (TCR) has, unfortunately, a long-standing history of high rates of registrations by death certificates only (DCO registrations). A study of all malignant neoplasms recorded by the TCR between 1987 and 1989 inclusive estimated that DCOs accounted for around 24% of all registrations (Pollock and Vickers, 1995). In 1992, the DCO rate was 20% (Thames Cancer Registry, 1995).

Each UK registry receives notification of all deaths occurring on its territory where cancer is mentioned on the death certificate. In the case of the TCR, about half of these patients identified from death certificates will already be known. The rest must be traced by following up case notes at local hospitals and treatment centres. Those cases not traced are defined as death certificate only registrations, or DCOs (Jensen et al, 1991). The TCR has attributed the high proportions of DCO registrations to the decision taken in 1983 (for financial reasons) not to follow up cases dying at home and to the amalgamation in 1985 into its territory of the North Thames region.

As the TCR contributes up to a third of England and Wales data, these high rates could bias regional and national survival analyses (Pollock and Vickers, 1994). (DCOs are excluded from survival analysis because it is rarely possible to confirm a date of diagnosis (Jensen et al, 1991).) They also cast doubt on the accuracy of regional and national incidence data because of a relatively high frequency of imprecision in certification of cause of death and data artefacts which can result in underreporting (Percy et al, 1981; Chow and Deveas, 1992; Gruhlich et al, 1995).

In recent years, the TCR has undertaken to reduce DCO rates. Analyses of FHSA data have increased ascertainment of cases seen outside National Health Service (NHS) acute settings. The registry has also gained access to the computerized information systems of some hospitals which had failed to provide manuscript case notes. Although these measures have helped to lower the proportion of DCOs, more dramatic reductions must be achieved if the TCR is to meet the requirements of the new Core Contract for cancer registries which came into effect in April 1996. The Core Contract sets standards of data quality that must be reached in the near future. For all cancers, DCOs should account at present for no more than 5% of registrations and a target rate of 2% has been set to be reached 'within 3 years' (EL(96)7, Annex A).

There is evidence to suggest that the largest reductions will be achieved by more effective ascertainment of cases seen in NHS acute hospitals. A study of factors associated with DCO registrations between 1987 and 1989 found that 40.5% of DCO cases died in NHS acute hospitals (Pollock and Vickers, 1995). This leaves out of account those cases seen in NHS hospitals who died else-

*Received 11 March 1998*
*Revised 14 January 1999*
*Accepted 22 February 1999*

*Correspondence to:* Am Pollock

**Table 1** Procedures in HES data

| OPCS code | Procedure<br>Endoscopy | OPCS code | Procedure<br>Surgery |
|---|---|---|---|
| Colorectal cancer (ICD-9: 153, 154) | | | |
| H20 | Endoscopic extirpation of lesion of | H04 | Total excision of colon and rectum |
| H21 | Other therapeutic endoscopic | H05 | Total excision of colon |
| H22 | Diagnostic endoscopic | H06 | Extended excision of right |
| H23 | Endoscopic extirpation of lesion of | H07 | Other excision of right hemicolon |
| H25 | Diagnostic endoscopic | H08 | Excision of transverse colon |
| H26 | Endoscopic extirpation of lesion of | H09 | Excision of left hemicolon |
| H27 | Other therapeutic endoscopic | H10 | Excision of sigmoid colon |
| H28 | Diagnostic endoscopic | H11 | Other excision of colon |
| | | H33 | Excision of rectum |
| | | H47 | Excision of anus |
| | | H48 | Excision of lesion of anus |
| | | Z94 | Laterality of operation |
| Cancer of the lung, trachea or bronchus (ICD-9: 162) | | | |
| E52 | Other operations on bronchus | E46 | Partial extirpation of bronchus |
| E54 | Excision of lung | E47 | Other open operations on |
| E55 | Open extirpation of lesion of lung | E48 | Therapeutic fibreoptic endoscopic |
| E57 | Other open operations on lung | E49 | Diagnostic fibreoptic endoscopic |
| E59 | Other operations on lung | E50 | Therapeutic endoscopic operations |
| Z94 | Laterality of operation | E51 | Diagnostic endoscopic |
| Female breast cancer (ICD-9: 174) | | | |
| B27 | Total excision of breast | B32 | Biopsy of breast |
| B28 | Other excision of breast | | |
| B29 | Reconstruction of breast | | |
| Z94 | Laterality of operation | | |

For all three tumour sites the procedure code for chemotherapy was X35. There is no code for radiotherapy.

where. In a case note study of colorectal cancer treatment in four districts covered by the TCR, case notes were retrieved on (58%) DCO cases registered by the TCR (Pollock and Vickers, 1994).

In view of this potential for a reduction of DCOs, we analysed a sub-sample of cases (all registrations for the three most common cancers, viz. colorectal, lung and female breast (ICD-9: 153/154, 162, 174)) to examine the extent to which TCR data could be linked to hospital episodes statistics (HES) data. These are data submitted to the Department of Health (DoH) by NHS hospitals on all the patients they admit. They are collected in the form of 'finished consultant episodes' (FCEs) – episodes 'where a patient has completed a period of care under a consultant and is either transferred to another consultant or is discharged' (Government Statistical Service, 1993). National returns of FCE data are not named and, as no unique identifier is used in England and Wales, they cannot readily be linked to other data sets such as cancer registries. This is one of the reasons national data have not been used before in this way.

In this paper we have attempted to overcome this problem through the use of probability matching (i) to match and link FCEs that appear to refer to individual patients; and (ii) to match these putative patients to patients listed in the TCR. We then considered some of the factors associated with successful HES/TCR matching and DCO retrieval in multiple logistic regression models.

Previous studies linking cancer registry data with electronic data have focused on pathology databases, and have used these to measure rates of ascertainment by the registry. We believe HES data are too crude to fulfil this aim. But they may be useful in tracing records that have not been included by the registry system.

### METHODS

Cancer registry data were requested from the Thames Cancer Registry on all residents of the Thames Regions (North and South) diagnosed with a malignant neoplasm of the colon, rectum, lung or breast (ICD-9: 153, 154, 162, 174) between 1 April 1991 and 31 March 1994 aged < 100 at diagnosis. Colorectal cancers were treated as a single category. Cancer of the lung refers to lung, trachea and bronchus. These data formed the reference data set.

HES data were requested from the Office of National Statistics (ONS) on all FCEs completed for residents of the Thames regions between 1 April 1991 and 31 March 1994 inclusive with a primary diagnosis of any of three cancers of interest aged < 100 at diagnosis.

Matching was carried out in two stages. The first stage used just HES data matching and linking episodes to different admissions that seemed to refer to the same patients. This was done using full date of birth, seven-character postcode and sex as index variables. Full accounts of the method and its assumptions have been published by Gill et al (1993) and Majeed and Voss (1995). The key assumption is that all FCEs with identical values in the index variables related to a single patient.

The second stage – using the same method – was used to match HES data to TCR data. Two matches were carried out sequentially, using slightly different matching criteria. The first (Match 1) was strictest: 3-digit ICD code, full date of birth (dd/mm/yyyy), sex and seven-character home postcode. The second (Match 2) was less strict: 3-digit ICD code, year *and* month of birth and the first four characters of the home postcode. Match 2 was carried out

*© 2000 Cancer Research Campaign* 

**Table 2** Number (%) of matched cases By tumour site and final source of cancer registration

| Match | All cases | Case notes | DCOs |
|---|---|---|---|
| Colorectal cancer (ICD-9: 153. 154) | | | |
| Match 1 | 12495 (63.5) | 11455 (69.3) | 1040 (33.1) |
| Match 2 | 1681 (8.6) | 1349 (8.2) | 332 (10.6) |
| No match | 5484 (27.9) | 3714 (22.5) | 1770 (56.3) |
| Total | 19660 (100) | 16518 (100) | 3142 (100) |
| Cancer of the lung, trachea and bronchus (ICD-9: 162) | | | |
| Match 1 | 13924 (51.9) | 11818 (57.6) | 2106 (33.4) |
| Match 2 | 2805 (10.5) | 2024 (9.9) | 781 (12.4) |
| No match | 10074 (37.6) | 6656 (32.5) | 3418 (54.2) |
| Total | 26803 (100) | 20498 (100) | 6305 (100) |
| Female breast cancer (ICD-9: 174) | | | |
| Match 1 | 13208 (56.7) | 12541 (60.4) | 667 (26.4) |
| Match 2 | 1975 (8.5) | 1711 (8.2) | 264 (10.4) |
| No match | 8106 (34.8) | 6506 (31.4) | 1600 (63.2) |
| Total | 23289 (100) | 20758 (100) | 2531 (100) |

**Table 3** Potential reductions in DCO rates by electronic matching

| Tumour site | DCO rates | | | |
|---|---|---|---|---|
| | Before matching | % | After matching | % |
| Colorectal cancer | 3142/19660 | 16 | 1770/19660 | 11 |
| Cancer of the lung, trachea or bronchus | 6305/26803 | 24 | 3418/26803 | 13 |
| Female breast cancer | 2531/23289 | 11 | 1600/23289 | 7 |
| All | 11978/69752 | 17 | 6788/69752 | 10 |

only on cases unmatched in Match 1. The potential reductions in DCO rates for the total three years were computed.

To test the significance of the association between HES/TCR matching and certain patient characteristics recorded by the registry, a backwards, step-wise, multiple logistic regression model was generated. Matching was the outcome variable (yes, no) and age group (< 75, 75 or older) and Regional Health Authority of residence were the explanatory variables. The strength of the association was measured by taking the change in deviance arising from the inclusion of each variable as an approximate $\chi^2$. All variables were modelled as categorical variables. The changes in the probability of matching associated with each variable were expressed as odds ratios.

The number of admissions was computed for matched DCO cases, stratified by tumour site and by the type of admission (ordinary in-patient, day case in-patient, and other) and the mode of admission (elective, emergency, other) recorded in the HES data set. HES coding defines variables according to specific criteria. Day-case admissions are in-patient admissions ‘given electively during the course of a day for care or treatment which can be completed in a few hours’. Regular day or regular night attendances of wards are not counted as day-case admissions. Elective admissions are planned admissions. Emergency admissions are defined as ‘admissions made at short notice at the request of Accident and Emergency services, general practitioners, bed bureaux or consultant out-patient clinics’. A third category, ‘other admissions’, also exists designating maternity FCEs and elective FCEs where a patient has been transferred from another health care provider (Government Statistical Service, 1993).

Procedures in the HES data were divided into four categories: (1) endoscopic, (2) surgical, (3) chemotherapeutic and (4) all others. The procedures in the HES data comprising category 1 and 2 are listed in Table 1. The number and percentage of patients to receive a treatment was computed by tumour site. Some common procedures for cancer, such as radiotherapy, are not classified in the standard Classification of Procedures used (OPCS 4).

### RESULTS

A total of 67 752 registrations of the chosen sites of cancer were identified in the TCR. Matches were found in the HES dataset for (66%) (Table 2). The proportion of cases retrieved for each tumour site was: 72% for colorectal cancer; 62% for cancer of the lung; and 65% for female breast cancer. For all three tumour sites the proportion of matches found for patients registered from hospital case notes was higher than the proportion found for patients registered as DCOs ($P < 0.0001$ for all three tumour sites). Among patients registered from hospital case notes, the proportions retrieved were: 78% for colorectal cancer; 68% for cancer of the lung; and 69% for female breast cancer. The corresponding proportions for cases registered as DCOs were: 44% for colorectal cancer; 46% for cancer of the lung; and 37% for female breast cancer.

If all of the matched DCO cases were followed up and case notes found for each, the proportion of DCOs for each tumour site would fall from 16% to 11% for colorectal cancer, from 24% to 13% for cancer of the lung, and from 11% to 7% for female breast cancer (Table 3). As a proportion of the total, DCOs would fall from 17% to 10%, equivalent to a reduction in the DCO rate of 43%.

 *© 2000 Cancer Research Campaign*

**Table 4** Multiple logistic regression model, factors associated with matching

| Variable | Sub-variable | No. matched/ no. sought (%) | $\chi^2$ | DF | Odds ratio |
|---|---|---|---|---|---|
| Age | <75 | 2217/4552 (48.7) | | | Reference |
| | 75 + | 3342/8916 (37.5) | 61.66 | 1 | 0.73 (0.67, 0.78) |
| Tumour site | Breast | 1209/3217 (37.5) | | | Reference |
| | Colorectum | 1385/3533 (39.2) | | | 1.20 (1.08, 1.32) |
| | Lung | 2965/6718 (44.1) | 56.02 | 2 | 1.43 (1.30, 1.56) |
| Region | S Thames | 2060/7386 (27.9) | | | Reference |
| | N Thames | 3499/6079 (57.5) | 1132.11 | 1 | 3.45 (3.21, 3.71) |

$P < 0.0001$ for all variables.

**Table 5** Number of admissions (number of patients) for matched DCOs by tumour site, type of admission and mode of admission

| Mode of admission | Type of admission | | Total | % |
|---|---|---|---|---|
| | Ordinary in-patients | Day case in-patients | | |
| Colorectal cancer (ICD-9: 153, 154) | | | | |
| Elective | 692 (452) | 346 (84) | 1038 (536) | 45.8 (37.1) |
| Emergency | 954 (661) | 3 (2) | 957 (663) | 42.2 (45.8) |
| Other[a] | 37 (13) | 234 (234) | 271 (247) | 12.0 (17.1) |
| Total | 1683 (1126) | 683 (320) | 2266 (1446) | 100 (100) |
| Cancer of the lung, trachea or bronchus (ICD-9: 162) | | | | |
| Elective | 1000 (588) | 424 (376) | 1424 (964) | 34.3 (31.3) |
| Emergency | 2315 (1749) | 4 (4) | 2319 (1753) | 55.7 (57.0) |
| Other[a] | 94 (49) | 310 (310) | 404 (359) | 9.8 (11.7) |
| Total | 3409 (2386) | 738 (690) | 4147 (3076) | 100 (100) |
| Female breast cancer (ICD-9: 174) | | | | |
| Elective | 624 (316) | 278 (92) | 902 (408) | 48.6 (40.7) |
| Emergency | 749 (419) | 9 (3) | 758 (422) | 42.0 (42.1) |
| Other[a] | 35 (10) | 162 (162) | 167 (172) | 9.0 (17.1) |
| Total | 1408 (745) | 449 (257) | 1857 (1002) | 100 (100) |

[a]Other: this category includes maternity FCEs and elective FCEs where a patient is transferred from another health care provider.

**Table 6** Number (%) of procedures recorded for matched DCO cases

| ICD Code | *n* | Endoscopy | Surgery | Chemotherapy | Other listed procedures | All procedures |
|---|---|---|---|---|---|---|
| 153/154 | 1372 | 238 (17.3) | 662 (48) | 33 (2.4) | 298 (21.7) | 1049 (76.4) |
| 162 | 2887 | 627 (21.7) | 219 (7.6) | 87 (3.0) | 461 (16.0) | 1514 (52.4) |
| 174 | 931 | 35 (3.8) | 194 (20.8) | 53 (5.7) | 207 (22.2) | 450 (48.3) |
| Total | 5190 | 900 (17.3) | 1075 (20.7) | 173 (3.3) | 966 (18.6) | 3013 (58.1) |

The results of the multiple logistic regression analyses are presented in Table 4. Diagnosis below age 75, tumour site (ranked by prognosis – with cancer of the breast which has the best prognosis as reference) and residence in North Thames region were all positively associated with successful matching ($P < 0.0001$ in all three cases). The factor that accounted for the greatest difference in the deviance of the models was tumour site.

For all three tumours sites, the largest single proportion of matched DCOs were admitted through emergency as ordinary in-patients (Table 5). Smaller proportions were also admitted for day case procedures. The admission : patient ratios for ordinary in-patient admissions were 1.5 for colorectal cancer, 1.4 for cancer of the lung and 1.9 for female cancer of the breast. The equivalent ratios for day-case admissions were 4.1,1.1, and 3.0.

The proportion of matched DCO cases admitted as 'other' admissions (i.e. neither elective nor emergency) is significantly greater than the proportion of all cases admitted by this mode: for matched DCO cases, 17% (colorectal), 12% (lung, tracheal or bronchial cancer), and 17% (female breast cancer) (not shown in a Table). The equivalent figures for all cases in the HES database were 1%, 2% and 1%.

A higher proportion of matched colorectal DCO cases had a surgical procedure (48%) and a lower proportion of lung cancers DCOs (8%) than was the case for cancer of the breast (21%). Few matched breast DCOs had an endoscopic procedure (only 4% compared with 17% and 22% for the other two sites). Overall, 76% of matched colorectal DCOs had at least one procedure compared with 53% for cancer of the lung and 49% for cancer of the breast (Table 6).

© 2000 Cancer Research Campaign

### DISCUSSION

Our study suggests that electronic linking of TCR and HES data might result in reductions of up to 43% in the proportion of cases registered as DCOs. The greatest reductions are likely to be made among the under-75s, among North Thames residents and among patients with tumours with poorer prognosis. There may also be a high proportion of patients transferred from other hospitals among the DCO cases. This is reflected in the higher proportion of patients admitted as 'other' admissions. The pattern of admissions found for DCO cases is distinguished from that of the HES database as a whole by the high proportion of patients admitted as 'other' admissions. This category comprises treated by maternity services and elective patients transferred from other hospitals. In view of the age and sex distribution of the data used for this study, it is unlikely that many cases would fall into the first category. We might expect it to be harder for registry clerks to trace notes on patients transferred from other hospitals since institutional responsibilities may become blurred in such cases; if so, then the high proportions of patients admitted for 'other' admissions among matched DCO cases might be a consequence of this difficulty. Further research is warranted to investigate this hypothesis.

It has been demonstrated elsewhere that DCO registration in the Thames regions is significantly associated with district of residence, old age, high tumour severity and dying at home (Pollock and Vickers, 1995). The associations with successful matching of DCOs detected here reflect these results.

The biggest reductions were achieved in relation to cancers of the lung, trachea or bronchus, the tumour site with the poorest survival rate and the highest proportion of DCOs. The lowest were found for female breast cancer, the tumour site with the best survival rate and the lowest proportion of DCOs. It is likely that the failure of follow-up and ascertainment is greatest among patients with more lethal cancers, since there will be less time in which to register them during life. The higher proportion of HES/TCR matched cases among North Thames residents might, likewise, be due to poorer cancer registration follow-up and ascertainment in that region. The lower proportion of matches among DCOs aged 75 or over may be due to the fact that a higher proportion of patients in this age group finish their care in residential homes. As their clinical case notes may go with them, they may be lost to the NHS and so not appear in the HES data. The earlier study found an interaction between age over 75 and patients finishing their care in residential homes (Pollock and Vickers, 1995). Further research is required to investigate all these hypotheses.

Fifty-eight per cent of matched DCO cases had at least one procedure recorded: 76% for colorectal cancer, 53% for breast cancer and 49% for female breast cancer. It should be borne in mind that these are minimum estimates. Some common procedures for cancer, such as radiotherapy, are not classified in the standard Classification of Procedures used (OPCS 4). There are doubts too about the assiduousness with which providers record treatments. The unit of contracting is the FCE, not the treatment; while there are financial penalties for failing to list FCEs, there are no incentives to account for treatments comprehensively. The classification system used to code procedures in the HES data set (OPCS 4) has no code for many treatments (Office of Population Censuses and Surveys, 1990); radiotherapy is among these. It is likely that some records with no procedures listed received a procedure labelled as 'other'.

### Record matching

Two sets of data-matching were carried out for this study. The pitfalls associated with the first stage (linking HES data to make them patient-based) have been discussed by Gill et al (1993) Three main sources of error can affect such matching but, in fact, are unlikely to have had much impact in the present study. The first stems from the assumption that records with the same ICD-9, seven-digit postcode, date of birth and sex relate to a single patient. Given the large spread of birth dates in our sample (such that few patients will share the same date) and the large number of postcodes (in comparison with the relative rarity of cancer in the population as a whole), the numbers of birth dates in our sample, and the large numbers of postcodes and the incidence of these cancers in the population as a whole, the probability of this assumption being false is very low. A more likely source of error is from coding mistakes. If there is inconsistency in the recording of any of the index variables, our method will assign the FCE to another patient record and this would lead to overestimation of patient numbers. However, since, in the case of DCOs, we are interested in the earliest hospital contact recorded, this should not affect the matching of cancer registry data with HES data. Lastly, the method of record linkage makes no allowance for patients moving home between FCEs. This could lead to an overestimate of *new* patient numbers. Again, however, the emphasis on the earliest contact should mean that this has little effect in the present study.

The second stage (matching cancer registry data to HES data) uses two sets of matching criteria. Both are extremely stringent (i.e. the probability of a valid match is close to 1), but here again mistakes in coding for any of the index variables will mean that true matches are missed.

The registry is currently examining proposals to move to electronic data collection through linkage to hospital database systems. Initially, matching would take place using named data (these are available at regional but not at national level). Thereafter, NHS number (a unique identifier) would be used. At present NHS number is not used by all NHS providers. In both cases, the proportion of successfully matched cases should be higher than those reported here. (The first stage of matching outlined here would not be necessary with named data.)

### CONCLUSION

Our experience of HES data suggests that the registry should proceed with care. HES data are less complete than cancer registry data. The lack of a specific code for procedures such as radiotherapy and the probable underreporting of procedures within FCEs mean that the registry should rely on HES data only for cases that have not been ascertained by the usual means, e.g. DCOs and here only as an aid to tracing records that have been missed by routine registry procedures. The many deficiencies of HES data mean HES data are a poor substitute for conventional cancer registry ascertainment. However, this study suggests that HES data can be used as an aid to the follow-up of known cases. A prospective study is required to establish the extent to which the potential reductions identified in this study are achievable in practice.

### REFERENCES

Chow WH and Devesa SS (1992) Death certificate reporting of colon and rectal cancers. *JAMA* **267**: 3028

*© 2000 Cancer Research Campaign*

Gill L, Goldacre M, Simmons H, Bettley G and Griffith M (1993) Computerised linking of medical records: methodological guidelines. *J Epidemiol Commun Health* **47**: 316–319

Government Statistical Service (1993) *Hospital Episode Statistics, 1989–1990*. HMSO: London

Grulich AE, Swerdlow AJ, Dos Santos Silva I and Beral V (1995) Is the apparent rise in cancer mortality in the elderly real? Analysis of changes in certification and coding of cause of death in England and Wales, 1970–1990. *Int J Cancer* **63**: 164–168

Jensen OM, Parkin DM, Maclennan R, Muir CS and Skeet RG (1991) *Cancer Registration: Principles and Methods*. IARC Scientific Publications: Lyon

Majeed FA and Voss S (1995) Performance indicators for general practice. *Br Med J* **311**: 209–210

Office of Population Censuses and Surveys (1990) *Classification of Surgical Operations and Procedures* 4th Edn. Office of Population Censuses and Surveys: London

Percy C, Stanek E III and Gloeckler L (1981) Accuracy of cancer death certificates and its effects on cancer mortality statistics. *Am J Public Health* **71**: 242–250

Pollock AM and Vickers N (1994) The impact on colorectal cancer survival of cases registered by 'death certificate only': implications for national survival data. *Br J Cancer* **70**: 1229–1231

Pollock AM and Vickers N (1995) Why are a quarter of cancers in South East England registered by "death certificate only" (DCO)? Factors related to DCO registrations in the Thames Cancer Registry between 1987 and 1989. *Br J Cancer* **71**: 637–641

Thames Cancer Registry (1995) *Cancer in South East England 1992*. Thames Cancer Registry: Sutton

© 2000 Cancer Research Campaign
